# Drip, Ship, and Grip, then Slice and Dice: Comprehensive Stroke Center Management of Cervical and Intracranial Emboli

**DOI:** 10.3389/fneur.2013.00104

**Published:** 2013-07-29

**Authors:** Jason D. Hinman, Neal M. Rao, Anil Yallapragada, Joe Capri, Puneet Souda, Julian Whitelegge, William H. Yong, Reza Jahan, William Quinones-Baldrich, Jeffrey L. Saver

**Affiliations:** ^1^Department of Neurology, David Geffen School of Medicine, University of California Los Angeles, Los Angeles, CA, USA; ^2^The Pasarow Mass Spectrometry Laboratory, University of California Los Angeles, Los Angeles, CA, USA; ^3^Department of Pathology and Laboratory Medicine, David Geffen School of Medicine at University of California Los Angeles, Los Angeles, CA, USA; ^4^Department of Interventional Neuroradiology, University of California Los Angeles, Los Angeles, CA, USA; ^5^Department of Vascular Surgery, University of California Los Angeles, Los Angeles, CA, USA

**Keywords:** stroke, stroke care, middle cerebral artery occlusion, carotid floating plaque, carotid endarterectomy revascularization

## Abstract

**Background and Purpose:** Tandem acute thrombotic emboli in the cervical and intracranial arteries are an unusual cause of stroke presenting unique management challenges. In regional systems of acute stroke care anchored by Comprehensive Stroke Centers (CSC), combined fibrinolytic, endovascular, and open surgical intervention is a new therapeutic option.

**Summary of Case:** A 28-year-old male underwent retinal surgery, including post-operative neck compression and the next day presented to a primary stroke center with aphasia and right hemiplegia. Intravenous tissue plasminogen activator therapy was initiated and the patient was transferred to a CSC for higher level of care (drip and ship). Imaging at the CSC demonstrated tandem thrombi: a near occlusive lesion at the origin of the left cervical internal carotid artery and a total occlusion of the M1 segment of the left middle cerebral artery. Endovascular thrombectomy with the Solitaire stent retriever resulted in intracranial recanalization (grip). Immediately after the endovascular procedure, open carotid thrombectomy was performed to achieve cervical carotid revascularization without systemic heparinization (slice). Both cervical carotid and intracranial thrombi were processed for proteomic analysis via mass spectrometry (dice).

**Conclusion:** Combined fibrinolytic, endovascular, and open surgical intervention can yield revascularization and good clinical outcome in cases of tandem lesions.

## Case Review

A 28-year-old Hispanic male with no significant past medical history had suffered a traumatic partial retinal detachment 2 weeks prior to neurologic presentation. On the day prior, he underwent retinal surgery at another hospital. Post-operatively, he was instructed to lay prone with face down in a circular head pillow to minimize retinal edema. After several hours in this position, he complained of neck pain but was deemed stable for discharge home. He went home and went to bed and awoke 2 h later with aphasia and right hemiparesis. He was taken to a nearby primary stroke center hospital. His exam was notable for dense right hemiplegia and non-fluent aphasia. Complete blood count, chemistries, and coagulation studies were all normal. Non-contrast head CT was unremarkable, and after telephone discussion with the comprehensive stroke center (CSC) team, he was administered intravenous tissue plasminogen activator starting 165 min after last known well time (drip). Transfer to the CSC was initiated (ship).

**Figure 1:**
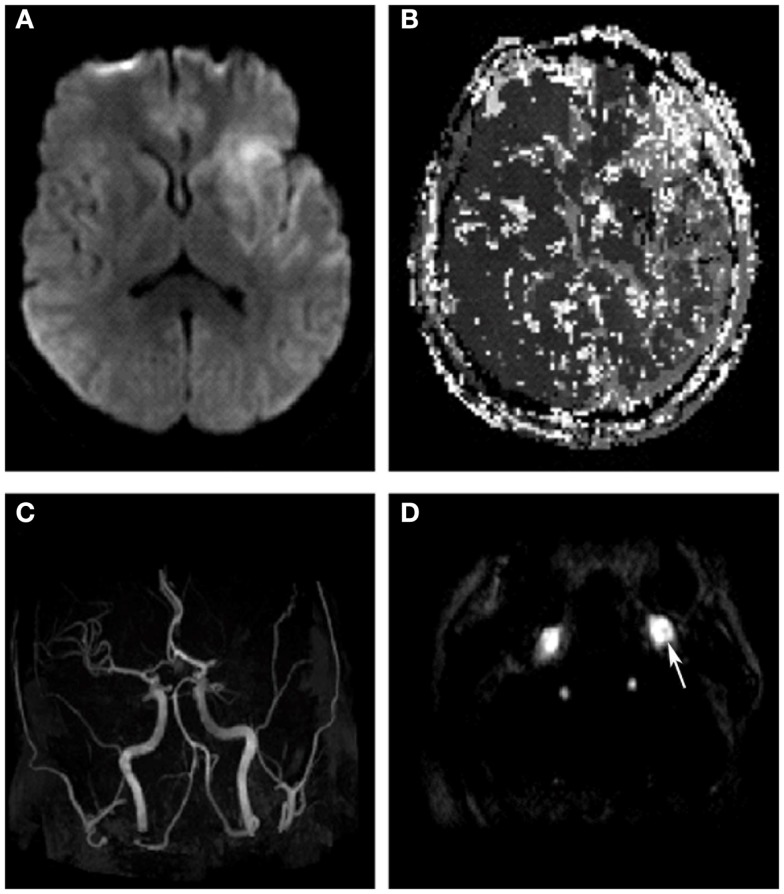
**Diffusion-weighted imaging shows left insular, frontal and superior temporal lobe early ischemic injury (A)**. Perfusion-weighted imaging shows delayed time-to-peak in the entire left middle cerebral artery territory **(B)**. Time of flight, MRA head shows abrupt cutoff in the left M1 segment **(C)**, while contrast-enhanced axial MRA of the neck shows a hypointense mass partially occluding the lumen of the left ICA (arrow)**(D)**.

**Figure 2:**
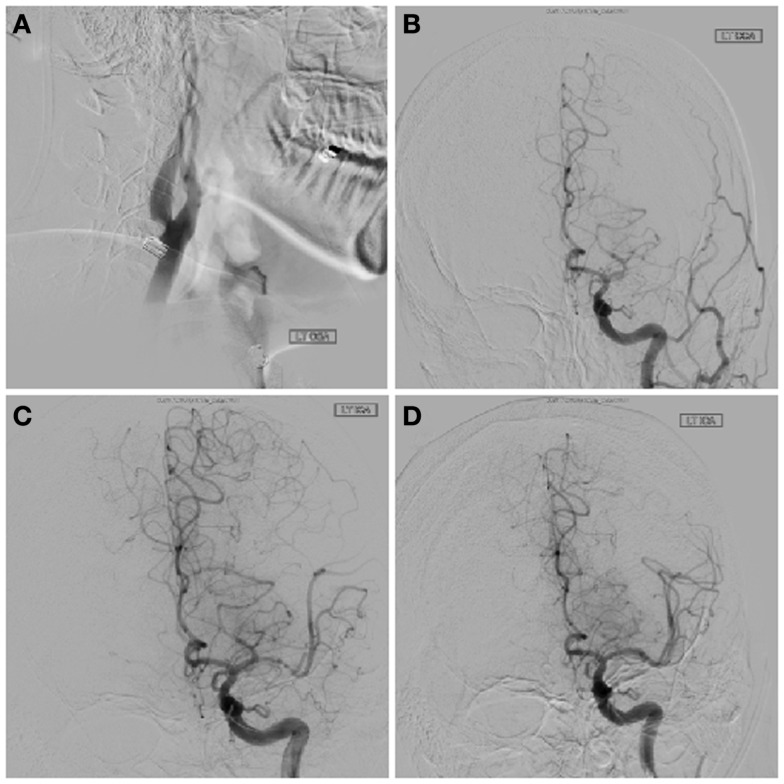
**Pre-treatment lateral angiogram of the cervical left ICA shows a focal thrombus attached to the posterior wall of the artery (A)**. Intracranial angiography confirms a left M1 occlusion on left common carotid artery injection **(B)**. Solitaire stent deployment partially improved distal flow **(C)**. Final TICI 2a angiographic result after embolectomy**(D)**.

Upon arrival, the patient had persistent aphasia and dense right hemiplegia, with NIH Stroke Scale of 24. Vital signs were remarkable only for elevated blood pressure at 150/87. MRI demonstrated diffusion abnormality in the left frontal operculum and superior temporal lobe with sparing of the primary motor and sensory cortices as well as Wernicke’s area (Figure 1A). Perfusion imaging was motion degraded but showed delayed perfusion to the posterior left frontal and parietal lobes (Figure 1B). MR angiography (MRA) showed two lesions: flow attenuation in the left cervical internal carotid artery (ICA) origin and occlusion of the intracranial proximal left M1 segment of the middle cerebral artery (MCA) (Figures 1C,D). The patient was taken to the neurointerventional suite for endovascular thrombectomy with groin puncture at 390 min after last known well time. Initial diagnostic digital substraction angiography demonstrated a sub-occlusive thrombus in the origin of the left ICA and complete left M1 occlusion (TICI = 0) (Figures 2A,B). With a guide catheter in the left common carotid artery and using a single pass of the Solitaire stent retriever (grip), the left M1 was successfully recanalized (Figure 2C), while mechanical maceration of a more distal M2 occlusion was performed with overall partial reperfusion achieved (TICI = 2a) (Figure 2D), 7 h after last known well time. Early post-intervention MRI 3 h after the procedure showed mild petechial hemorrhagic transformation of the infarct in the left basal ganglia and the patient was admitted to the neurologic intensive care unit (NICU) for monitoring with minimal change in the neurologic exam.

**Figure 3:**
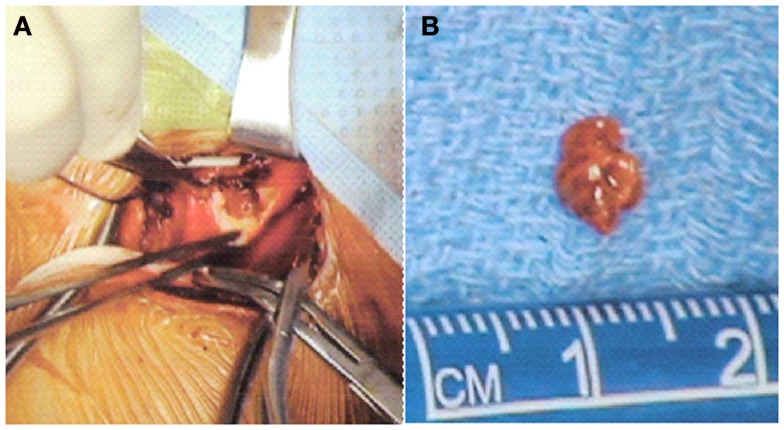
**Intra-operative images showing the cervical lesion *in situ* in the left internal carotid (A) and the 5-mm thrombus after operative thrombectomy (B)**.

**Figure 4:**
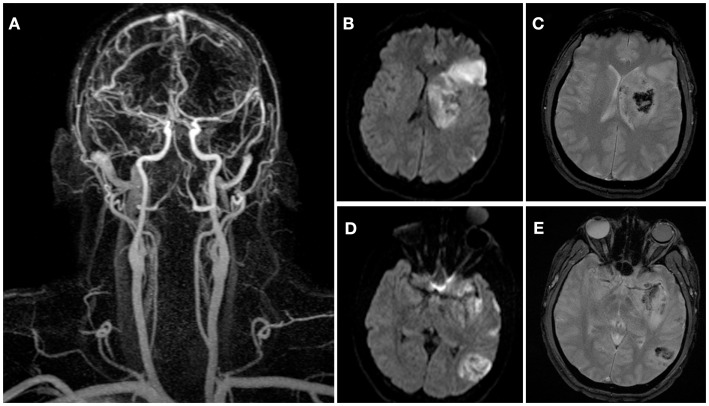
**Contrast-enhanced MRA obtained 24 h after stroke onset demonstrates final angiographic result with patent left cervical carotid and middle cerebral arteries (A)**. Post-intervention 24 h followup diffusion-weighted **(B,D)** and gradient echo **(C,E)** images showing the final infarct volume (41.7 vs. 37.7 cc, initially) and mild hemorrhagic transformation of the lesions.

The sub-occlusive thrombus at the origin of the left ICA was deemed too risky for endovascular treatment either via endovascular stent placement or suction thrombectomy due to its large size and high risk of distal embolization. Because the risk of distal embolization to the brain was also considered substantial with medical therapy, vascular surgery was consulted and open surgical thrombectomy pursued. Due to the mild post-reperfusion hemorrhagic transformation in the cerebrum, standard systemic heparinization during thrombectomy was deemed unsafe. An open thrombectomy without systemic heparinization was performed 11 h after last known well time (slice). A small soft clot was removed from the origin of the left ICA with only mild atherosclerotic changes underlying the thrombus (Figure 3). The surgical field was irrigated with heparinized saline, the carotid artery was closed, and the patient was returned to the NICU for further care. Post-operative transcranial Doppler revealed only one high intensity transient signal in the left MCA, while repeat MRI at 24 h post-intervention revealed no evidence of re-thrombosis or progression of the intracranial hemorrhage (Figure 4).

**Figure 5:**
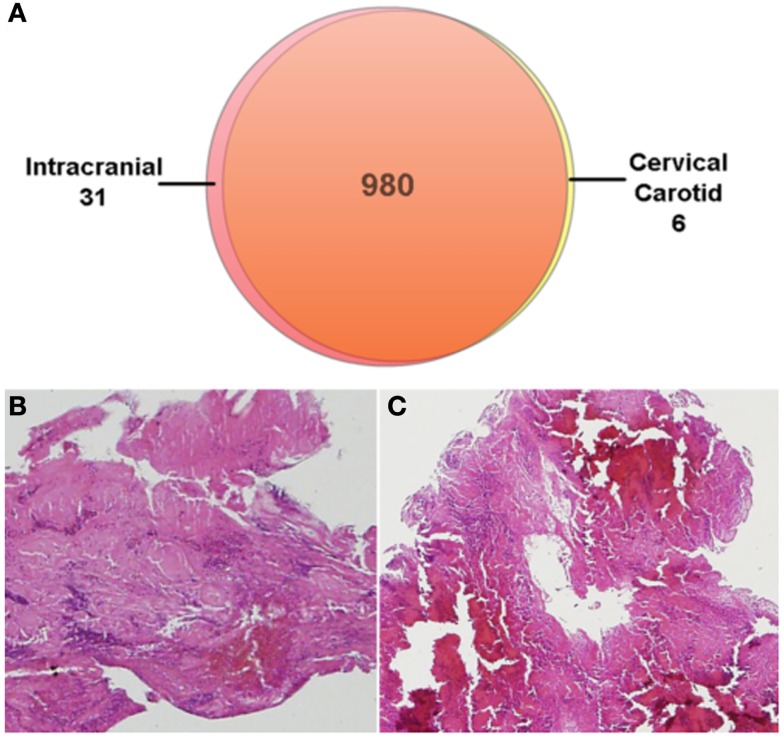
**Proteomic analysis of the retrieved thrombi revealed 980 common proteins (A)**. Six proteins were unique to the cervical carotid thrombus while 31 proteins were unique to the intracranial thrombus. Hematoxylin and eosin stain of the intracranial **(B)** and cervical **(C)** thrombi.

As part of an ongoing IRB-approved research study, both the intracranial thrombus retrieved using endovascular thrombectomy and the carotid thrombus obtained during open surgical thrombectomy were frozen and processed for mass spectrometry proteomic analysis (dice). Histopathologic and proteomic analysis of the thrombi were performed as part of an ongoing study to determine the origin and composition of stroke-causing thrombi. Liquid chromatography-mass spectrometry revealed 980 proteins common to both thrombi, 6 unique to the cervical carotid thrombus, and 31 unique to the intracranial thrombus (Figure 5).

Diagnostic evaluation over the next several days included negative tests for hypercoagulable states and an unremarkable transesophageal echocardiogram with bubble study. Lipid profiling was abnormal with an LDL of 172 and triglycerides of 299. The patient’s ESR was slightly elevated at 19 and his CRP was markedly elevated at 21.4. A presumptive diagnosis was made of traumatic thrombosis due to positional compression of the carotid artery. He was discharged to acute rehabilitation 10 days after admission with residual right hemiparesis and mild transcortical motor aphasia, NIH Stroke Scale 15. The modified Rankin Scale was 5.

## Discussion

Compression of the carotid artery is a rare but well-described cause of local thrombosis and stroke. Case reports of carotid compression, thrombosis and stroke have been reported after a patient fell asleep on his elbow, sexual aphyxisation, and blunt trauma (1–3). In this case, we suspect that the post-operative prone period resulted in carotid compression, local thrombosis, and distal embolization leading to stroke.

The optimal management of tandem cervical and intracranial thrombi in patients with acute cerebral ischemia has not been well-delineated in the literature. Substantial experience has been gained with the more common combination of a cervical atherosclerotic lesion with supervening *in situ* thrombosis and distal daughter intracranial embolization. For that combination, the intracranial lesion can be addressed by fibrinolysis and/or endovascular thrombectomy and the cervical lesion by acute angioplasty and stenting or delayed endarterectomy or carotid stenting. However, when the cervical lesion is entirely or predominantly thrombus rather than atherosclerotic plaque, this approach carries substantial risk. Immediate cervical carotid angioplasty with or without stenting has a high risk of causing further embolization by dislodging the large thrombus. Delayed endarterectomy carries a high risk that early re-embolization from the cervical carotid to the intracranial circulation will occur under initial medical therapy while waiting for sufficient recovery from stroke to permit surgery.

The approach taken in this patient of acute surgical cervical thrombectomy immediately following systemic fibrinolysis and intracranial endovascular thrombectomy has not been previously reported to our knowledge. Compared with carotid endarterectomy for atherosclerotic plaque, surgery for thrombosis can be performed more quickly and with less disruption of the endothelial surface. In this case, the need to avoid intra-operative anticoagulation was a particular concern due to the early minor hemorrhagic transformation (4, 5), but the risk of re-thrombosis was considered low in a young patient with relatively normal underlying endothelium.

This case also illustrates the successful application to a young stroke patient of a regional spoke and hub system of ischemic stroke care, as endorsed by the Brain Attack Coalition (6) and the American Stroke Association (7). Initial transport to a Primary Stroke Center permitted rapid initiation of intravenous fibrinolysis and prompt interfacility transfer to a CSC permitted timely delivery of a complex, multidisciplinary intervention guided by advanced neurovascular imaging and the availability of multiple specialists. Proteomic analysis of thrombi obtained from patients presenting with acute stroke offers the opportunity to investigate thrombotic mechanisms that may be unique to stroke. Such patient-centered research activities are one of the core elements required for CSC certification (7).

## Conflict of Interest Statement

The authors declare that the research was conducted in the absence of any commercial or financial relationships that could be construed as a potential conflict of interest.
